# Socio-cultural reflections on heat in Australia with implications for health and climate change adaptation

**DOI:** 10.3402/gha.v5i0.19277

**Published:** 2012-10-16

**Authors:** Cathy Banwell, Jane Dixon, Hilary Bambrick, Ferne Edwards, Tord Kjellström

**Affiliations:** 1 National Centre for Epidemiology and Population Health, The Australian National University, Canberra, ACT, Australia; 2 Centre for Health Research, University of Western Sydney, Campbelltown, NSW, Australia

**Keywords:** older Australians, experience of heat, health, climate change, vulnerability, impacts, adaptation, Australia

## Abstract

**Background:**

Australia has a hot climate with maximum summer temperatures in its major cities frequently exceeding 35°C. Although ‘heat waves’ are an annual occurrence, the associated heat-related deaths among vulnerable groups, such as older people, suggest that Australians could be better prepared to deal with extreme heat.

**Objective:**

To understand ways in which a vulnerable sub-population adapt their personal behaviour to cope with heat within the context of Australians’ relationship with heat.

**Design:**

We draw upon scientific, historical and literary sources and on a set of repeat interviews in the suburbs of Western Sydney with eight older participants and two focus group discussions. We discuss ways in which this group of older people modifies their behaviour to adapt to heat, and reflect on manifestations of Australians’ ambivalence towards heat.

**Results:**

Participants reported a number of methods for coping with extreme heat, including a number of methods of personal cooling, changing patterns of daily activity and altering dietary habits. The use of air-conditioning was near universal, but with recognition that increasing energy costs may become more prohibitive over time.

**Conclusions:**

While a number of methods are employed by older people to stay cool, these may become limited in the future. Australians’ attitudes may contribute to the ill-health and mortality associated with excessive heat.

Over the last couple of decades, there have been a number of dramatic heat waves across Europe, the United Kingdom, the United States, Asia, and Australia that were associated with significant mortality. This article examines Australians’ relationship to extreme heat and looks at how a group of more vulnerable people (elderly) respond to heat waves. Thus far, climate change research has been dominated by the natural sciences and economics. What is needed is input from the interpretive social sciences to ‘shed new light on the multiple meanings of climate change’ (1). Here we respond to this challenge by drawing on historical, literary, and scientific sources to reflect on manifestations of Australians’ ambivalence towards heat and heat waves and how we respond behaviourally to hot weather.

Australia is a vast country that ranges from a tropical climate in the north, extremely hot deserts in the centre, to temperate coastal regions in the south, east, and west (Fig. 1). From the beginning of the Europeans’ settlement in Australia, its climate, particularly heat and drought, has been a source of concern and wonderment to new arrivals: something to be celebrated or endured. Australia provided settlers with climatic experiences that were vastly different from those they were familiar with and, in this way, heat has played a fundamental part in influencing patterns of population settlement for more than 200 years.

**Fig. 1:**
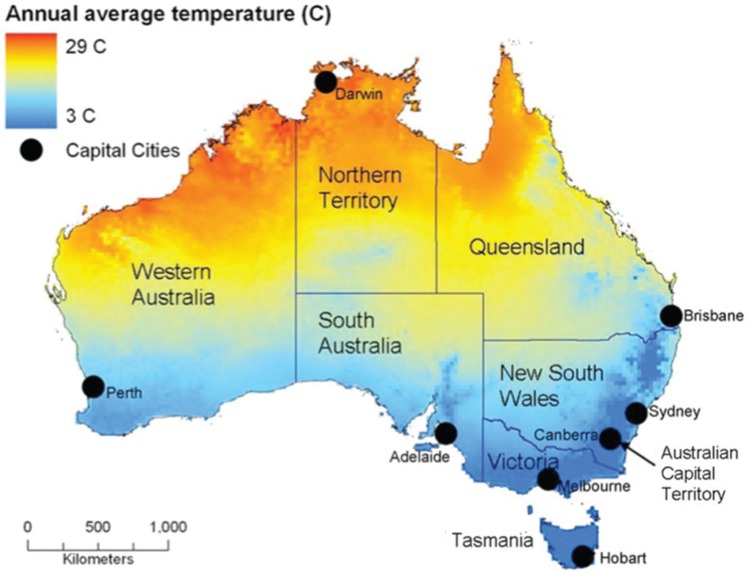
Average annual temperatures in Australia, showing states, territories, and their capital cities.

We argue that Australians’ cultural acceptance of heat is sedimented within the national psyche: the sun, the sea, and the outdoor barbecue reappear over and over again as iconic Australian scenes. In short, there are good reasons as to why Australians have developed a culture with an ambivalent relationship to the sun's heat. This can be expressed in the following quotation from the first decade of the 20th century.

*To the fierce sun of Australia, which tempers men as fire tempers steel; to the gracious sun of Australia, which makes nature teem with bounty; to the glowing sun of Australia, which warms the heat, enkindles the eye, ruddies the cheek, this in the tribute* (2)

In the late 19th century, it was thought that racial characteristics and even political systems were determined by climate. Following this logic, Stefansson proclaimed that ‘colder climates produced more complex and highly evolved civilizations than tropical ones’, partly because of enervating heat and partly because the tropics produced food so easily that strong, fit bodies were not required (3). Heat was seen at this time as a major problem for Australians and some believed that pioneer Australians ‘lacked vim’ and ‘showed signs of having been drained by their climate’ (cited in 3: 94).

Sunstroke was believed to cause madness for the colonial white male. In the 1870s, 16% of the male patients in the Yarra Bend asylum near Melbourne were identified as ‘sunstruck’, leading many to believe that the Victorian colony was in the ‘most unenviable position of being the maddest place in the world’ (4). Boucher (5) likens this malady within the medical discourse of the time as a consequence of white, middle-class, male, protestant bodies being out-of-place with the Australian environment and climate.

However, rather than admit that they were unable to cope with the climate, settlers were determined to make Australia work for them, embracing and celebrating the resilient and renewed character of the Australian Bushman (those living in remote areas colloquially termed ‘the bush’). The arguments about heat were turned around and instead heat was seen as a positive force in fostering strong, resilient and healthy people.

These European beginnings were suffused with attitudes of control and progress that informed the development of the white Australian psyche. The attempt to control nature and the weather continued through technological fixes, such as irrigation systems, dams, bores, the planting of forests, and the seeding of clouds (6), and modern ‘climate-controlled’ cars, refrigerators, and air conditioners. For these reasons, Lowe (7) suggests that European settlers and their descendants have never truly engaged with the Australian continent and climate, instead imposing characteristics and behaviours from their previous lives in cooler climates.

## City life

Despite the strength of the ‘Aussie Bushman’ image, Australia is one of the most urbanised countries in the world. Ninety percent of Australia's population is concentrated along the coast mainly within its six State capital cities (8). As Australians have moved from the bush to the cities, so too has the national identity shifted. In city settings, the Aussie Bushman transformed into the Aussie Battler (or working-class Australian) who has developed an appreciation of warmth and the outdoor life and has channelled the battling spirit into sporting activities; success at which indicates national strength and determination.

The move to the cities was also a move to the coast. The Australian coast has a long history of inhabitation, serving as a healthy holiday destination since the era of the First Settlers, where the combination of sea breeze and sun acted as a cure-all and a place for renewal from the grey British skies. The beach and its climate became regarded as a symbol of the ‘new Australian's’ adaptability and endurance, where the Australian heat was seen as energising – Australian heat created ‘bolder and more adventurous characters and more resilient bodies’ (2: 97). Furthermore, Australian bodies were toughened by engagement with rough seas. The sun as a source of pleasure and revitalisation became fully realised in the surf culture, which flourished on the wave of consumerism and bikini contests in the 1920s and 1930s. The beach, and surfing in particular, took on the role of the urban equivalent to Australia's frontier, according to Fox (2) writing at this time. The coast offers a gentler climate than inland, access to cooling sea breezes, and a mechanism for Australians to be ‘sun-lovers’ while escaping the harshest heat of the inland. Despite Australians’ claims to toughness in the face of extreme climate, the beach is sought after and valued as a respite, a pleasure, and the site of hobbies, such as fishing, swimming, surfing, walking, and sunbathing.

Unremarkably, heat has profoundly influenced many aspects of Australian life although Australians have retained an ambivalent attitude to it. It is accepted as part of the Australian way of life and integral to the habitus. Unlike cooler climate locations where heat waves have caused a huge spike in deaths, heat waves are not considered ‘anomalous’ events in Australia (9). This sanguine disposition was a problem during a 2009 heat wave in Adelaide (South Australia) when there was 10-fold increase in sudden deaths, mainly among the elderly, in one day (10).

Australian meteorological records support the case for climate change. Average land temperatures have increased in Australia over the last half century and are predicted to increase by a further 1°C by 2030. The southern half of the Australian continent is no stranger to long-term heat accompanied by droughts, which present a range of health, economic and environmental impacts. Rural droughts and urban heat waves differ in terms of the number of people affected, and in the duration and intensity of exposure, resulting in a range of impacts on peoples’ health and on the environment. This article focuses on heat in urban areas where the dominant population of Australia resides and where the ‘Urban Heat Island Effect’ (11) exacerbates heat stress as the city's topography and urban infrastructure store and retain heat exposing urban populations to on-going, high temperatures (12).

## Heat and health

Australians’ cultural acceptance and ambivalence towards heat has resulted in it not being considered an important topic of research until recently. A formal review of literature published between 1998 and 2009 found that most research had been undertaken overseas with particular reference to the recent Chicago and European (mainly French) heat waves. However, historian Janet McCalman describes the heat wave of the late 1800s in Melbourne in which up to 50% of infants from poorer families died. At this time, there was a hot wind, flies were prevalent due to lack of sewage system, houses were un-insulated, water supplies were intermittent and babies were swaddled (13). The night air was feared as a bearer of disease so people did not open their windows (14).

Heat waves have multiple definitions in Australia. They are considered as periods of anomalous heat that generate a societal response (9) or as ‘a period in which the daily maximum temperature is equal to or greater than 35°C for three or more consecutive days’ (15). In 2009, Melbourne endured three consecutive days of temperatures above 43°C culminating at 45.1°C shutting down transport and the electricity systems, resulting in 374 excess deaths or a 62% increase in total all-cause mortality (16). Similarly, extreme heat waves and corresponding mortalities have been experienced in the cities of Adelaide and Sydney. According to the Australian Bureau of Meteorology (17), heat waves have actually accounted for more deaths in Australia than any other natural hazard. Increasingly the links between climate change and health are being investigated (18–20), with some pointing to heat waves as potentially one of the most damaging side-effects of global warming and proposing that the death rate from heat waves will triple by 2100 in all Australian cities (21).

**Fig. 2:**
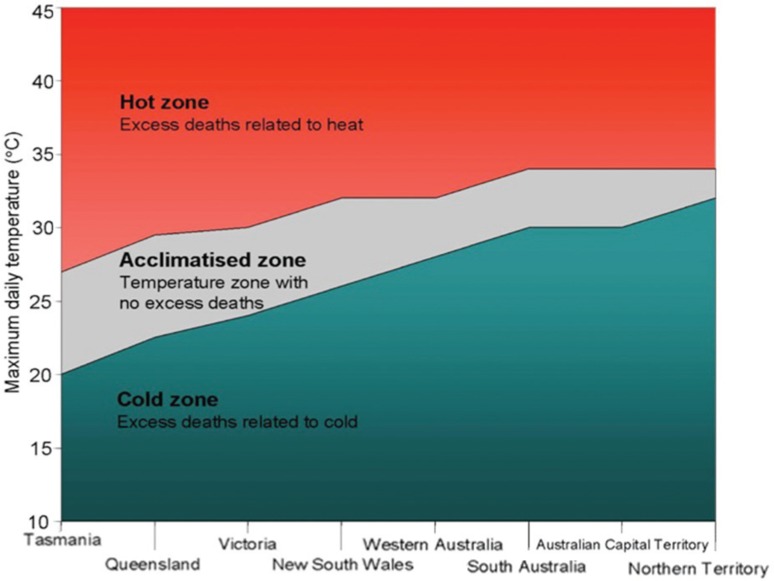
Temperature–mortality zones for Australian states and territories. In the ‘hot zone’ (red), the relative risk of death is increased with higher daily maximum temperatures, whereas relative risk of death is increased with lower maximum daily temperatures in the ‘cold zone’ (blue).

However, heat-related mortality is complex and thresholds at which all-cause mortality increases differ by location; this is most likely due to a level of acclimatisation in the local population (22) (Fig. 2). Overall, the risk of death is likely to be a combination of local biophysical, structural, and behavioural adjustments alongside underlying population characteristics, such as levels of chronic disease.

Limited research exists on the impacts of heat waves in the Australian cities of Melbourne (23, 24), Sydney (25), Adelaide, and in South Australia (15, 26–28). These articles s identify vulnerable people as those who are elderly or very young, who have coronary disease, mental health issues, or who are poor. ‘Vulnerability’ is defined as the degree to which people are sensitive to or have the adaptive capacity to cope with heat conditions (9). Many of the health conditions exacerbated by heat waves are triggered at a range of temperatures, that is, the prolonged nature of a heat wave results in deaths with people dying on the second or third consecutive excessively hot day (29). People's vulnerability to extreme heat also relates to the range of temperatures that people are familiar with where they live indicating ability to acclimatise to unusual weather conditions. The elderly are particularly vulnerable to heat as their thermoregulatory function declines with age and their hearts are less able to cope with increased demands. Furthermore, many of the medications that the elderly are prescribed interfere with the body's response to heat (30).

Heat kills in two ways: it either exacerbates pre-existing illnesses or produces heat-specific illnesses. Individuals with pre-existing, chronic medical conditions, such as heart and pulmonary disease, diabetes, alcoholism, and spinal-cord injuries are particularly vulnerable to heat waves. People with these conditions are often elderly (defined here as 60 years and older) (31, 32). Direct heat-related illnesses progress in three stages: heat stress, heat exhaustion, and heat stroke. Heat stress is not caused by heat alone but by a combination of humidity, wind, and air pollution (25) over a period of time, while social and political factors also play a significant role (30).

Green et al. (28) identified a combination of other heat-induced deaths which include lack of familiarity with harsh Australian conditions, the wearing of excessive clothing, acute alcohol intoxication, certain types of medication, and obesity. It is not just the physical manifestation of heat-related illness that is a concern. There is evidence (27) that temperature above 26.7°C increases hospital admissions for symptomatic mental disorders, including dementia and mood disorders. Excessive heat and humidity compounded by factors of lethargy, lack of sleep, and the inability to function normally may affect mood and behaviour, increasing mental stress, depression, and suicide, and triggering irritability and risky behaviours such as excessive alcohol consumption, violence, and aggression. Alston and Kent (33) suggest that depression and suicide among Australian farmers during drought is exacerbated by the historical image of Australian men as rugged and resilient individuals, who do not seek support, with a stoic resistance to adversity. Stemming from historical events is a culturally constructed need to control surroundings and failure to do so in times of enduring drought is seen as personal failure (33, 34). Although city dwellers are protected from the immediate effects of droughts, newspapers frequently report on the falling capacity of city dams and are required to reduce their water consumption with heavy penalties for non-compliance.

This article argues that cultural attitudes to heat developed over the last 2 centuries by European descended (non-indigenous) Australians continue to shape both everyday practices and research on heat. In turn, these attitudes influence Australians’ responses to climate change and adaptation to potential new extremes. As Dessai et al. (35) point out, a substantial gap exists between scientific definitions of climate change and an understanding of what constitutes dangerous climate change, particularly in the context of individuals’ everyday lives. In everyday terms, individuals need to reflect on whether climate change is occurring (or not), whether it is caused by human activity or not, whether it is hazardous, and whether it affects them personally. Research on extreme heat impacts is dominated by quantitative research, and we are unaware of any other studies that have looked at what people actually do to minimize the discomfort and health risks associated with heat. To complement the literature on the scientific, social, and health experiences of heat in domestic settings, we undertook a small exploratory qualitative study with a group of especially vulnerable people – elderly residents of Western Sydney. It takes a phenomenological approach to interrogate ordinary and everyday practices by people in their home environments when faced with high temperatures.

## Methods

**Fig. 3:**
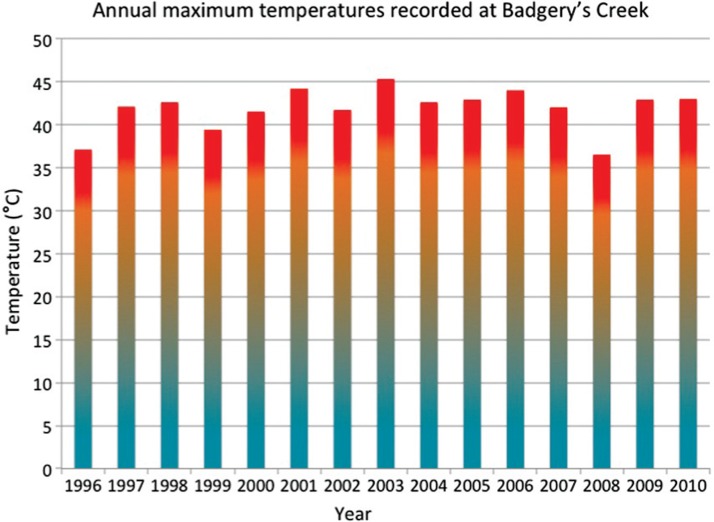
Annual maximum temperatures recorded at Badgery's Creek, at the centre of the study region.

Western Sydney is an area of rapidly expanding suburbs on the inland side of Sydney where green space and trees are being replaced by new commercial and domestic developments. As it is inland and not tempered by coastal climate, average temperatures in Western Sydney can be high in summer (Fig. 3).

Research participants were recruited through the University of Western Sydney's community engagement service for medical research. Volunteers were sought to take part in a qualitative study on heat and health and a pamphlet was circulated. We obtained permission to telephone volunteers to establish a date and location for interviews. The first set of interviews was carried out in early spring (September) 2008 and repeat interviews with a number of the same respondents were conducted in the middle of summer (January) 2009 so that we could see if the effect of immediate heat made any difference to the interview content. Two interviews were conducted with two older men and four women (and included two couples). In addition, we obtained detailed written responses to our interview questions from a man and a woman who were not well enough to sit through a lengthy interview. We also conducted two focus groups with mainly female residents of an aged-care facility, each lasting approximately 2 hours. Both the individual interviews and the focus groups were semi-structured, with specific items included that were then followed up to elicit further and more detailed information. Where possible, interviews were conducted in participants’ residences and we noted the layout, organisation and the strategies used to manage heat.

Interviews were conducted by three of the authors (CB, JD, and HB) in participants’ homes or at the aged-care facility. Participants were asked about the influence of heat on their daily activities, including eating patterns and food choices, sleeping patterns, social activities, domestic tasks, use of transport, how they managed their environment, and the perceived effects of heat on their health. Finally, they were asked whether they thought that their experiences of heat were related to climate change and global warming. The interviews lasted about an hour and were digitally recorded and transcribed verbatim by an independent service. A coding framework that aimed to capture the obvious and underlying meanings of the text was developed by repeated reading of the interviews and, due to the small number of interviews, they were coded and analysed by hand. Ethical clearance was obtained from the ANU Human Research Ethics Committee (approval number 2008/353).

## Results

A total of eight participants were individually interviewed and 12 participated in the focus groups. Six of the original interviewees were followed up for a second interview. All participants were aged over 65, were either on a pension, or a pension with superannuation, and reported household incomes of less than AU$30,000 per annum. More than two-thirds of the participants were women. Participants lived in a range of dwellings, including a retirement village, an aged-care facility (supported care), half a house, and a converted garage. Pseudonyms have been used for participants to protect their privacy.

### Living with heat

Even in this small sample, there was a range of attitudes and practices in response to heat. There were no notable differences between the responses from the men and women in the study. However, all participants agreed that they changed their daily patterns of living when it became hot. Most commonly, they altered and reduced their physical activity levels. For example, one woman said that she *did not play as much tennis* when the weather was hot while others said they *did nothing* during the hottest parts of the day. Jessica noted that her routine changed on very hot days.

*Like usually I wake up very early, but if it is hot I don't go out … So I do some paperwork. I tidy up and then I just stay indoors*

Because participants were aware of the positive health effects of maintaining physical activity, they sometime chose to do gardening, walking, and housework in the early mornings (as early as 4 am) rather than giving up completely in hot weather. One couple, Ted and Alicia, had adopted air-conditioned ‘mall-walking’ for about 45 min a day as a way of maintaining exercise irrespective of the temperature and season and as a result had lost quite a lot of weight. Rose differed from others because she *didn't mind the heat* and had a swimming pool at home. She said her routine did not change much but she wore her swimming suit all day and dipped in the pool frequently on hot days.

### Food

When it was hot, participants said they ate *lighter* foods, which often included salads, ice-cream, and other cold foods. Some thought that cooking on a barbecue and eating outside were enjoyable aspects of summer. They drank more fluids, although none reported consuming more alcohol, even though beer is promoted in advertisements as a way of coping with heat and thirst. Some noted that they lost a little weight during summer because of dietary changes. Jessica who was less well-off, differed from other participants because she lived in a small space where her stove heated her room if she used it. She was therefore reluctant to cook and ate more take-away and pre-prepared foods and was concerned about her weight.

### Social activities

Participants’ face-to-face social connectivity was reduced in hot weather and they said they felt *couped up*. However, they all had strong social networks and they kept in touch with friends and relatives by phone. A common problem was difficulty getting to sleep and *tossing and turning* when it was hot.

### Cooling methods

Most participants had an air-conditioner or fan in their bedroom. Jenny explained:

*A couple of years ago I bought a fan for above the bed because I just couldn't sleep. [Before] I had been bringing a yoga mat out and sleeping out here [the living area] with the air-conditioning to keep cool*.

All participants had an air-conditioner somewhere in their dwelling and in their car, if they drove one. One couple commented that as *we got older we found we need it and we put in reverse cycle air-conditioning*. They used them frequently but also supplemented them with other strategies for managing their home environment. Air-conditioners in the house and car were seen as the major strategy for managing heat although they were used judiciously to reduce electricity bills. Rose, who lived in the top half of a house observed:

*We only have [air-conditioning] on at very limited times, because in the afternoon in the summer there is a lovely breeze*.

They opened and closed doors and windows to catch cool breezes and used curtains and other methods to block direct light, and a number of people used ceiling fans as well. Water was used to cool off; couples with a swimming pool used it frequently while others had showers or cool baths or just splashed water on the face. Several had insulation installed in the ceiling as well. While the air-conditioner clearly trumped other heat management strategies, participants remembered times past when few people had them and instead peopled placed wet tea towels in front of fans or sucked ice or had played in sprinklers as children.

It was more difficult for people to heat-proof their external environment; one couple lived in a retirement village and the garden was managed centrally. Others noted that there were not many trees in their suburbs, making the urban landscape hotter and, like many Australians, they lived in modern houses without eaves or verandas. To compensate for their lack of control over the exterior, participants relied on managing their interior temperatures.

If they could afford it, they travelled. One couple were typical ‘grey nomads’, a colloquial term to describe older, often retired people who take extended trips by road. They would travel north to warmer temperatures during winter and then take their caravan to the beach during summer. Another couple timed their overseas trips to avoid the cold. Indeed, cold and its effects on the body were considered more unpleasant by participants than heat.

### Health problems

Primary health effects of climatic change have been identified as those that have a direct effect on human health (36). As we have already noted, the literature on the acute health effects of heat finds that heat exacerbates pre-existing illnesses and produces heat-specific illnesses, particularly in the elderly. Elderly individuals with pre-existing, chronic medical conditions are especially vulnerable to heat waves (32). Participants in this study observed that heat made their existing health conditions worse with one woman suffering extreme migraines when it was hot. Generally, heat was associated with lack of energy and other complaints, as interviewee Francis noted:

*I find [humid heat] very de-enervating … A couple of times when I've been out when it has been warm and you go to do a little bit of bending over or something or other you get a bit dizzy but the doctor tells me that would be because in the heat, your blood pressure drops a little*.

Another woman commented that being overweight and hot and elderly was a trial and further noted that being overweight *when it's hot, your legs rub together and you chaff and it's sore and it's uncomfortable*.

This unpleasant effect of heat had in fact been a motivating factor for her to take action to lose weight.

At various times in their lives, participants had suffered sunburn, felt faint or dizzy but none had experienced more severe problems. During periods of extreme heat, they followed health advice (which they heard on the radio rather than from their doctors) such as avoiding the sun during the hottest time of the day and drinking lots of water.

As yet research on climatic change and human health has not considered the more distal effects of heat on major lifestyle-related conditions, such as obesity, diabetes, and hypertension. These interviews suggest that an increasing frequency of heat waves over time may reduce activity levels, alter dietary patterns, make sleeping more difficult, and reduce face-to-face connectivity, thus possibly impacting on both physical and mental health.

### Climate change

Some participants dismissed the notion that climate change and global warming existed and they did not connect it with the last few years of unusually hot, dry weather. Instead, they proposed that they felt it was hotter now because they were older or they did not think that it was any hotter than in the past. Rose said:

*I can remember when I was only very young and it was so very hot. … I remember when babies died in hospital because it was so hot*.

The only people who acknowledged the possibility of climate change were Ted and Alicia, the grey nomads, who attributed it to a wide range of forces.

*I think it is essential that we all become aware of our impact on the planet and climate. I also think that most negative effects on the climate and the planet are caused by irresponsible science, governments, and production of weapons of mass destruction*.

For these older Australians, the prospect of hotter temperatures associated with climate change did not motivate them to modify their daily practices as they either did not ‘believe’ in it or feel that they contributed to it.

## Discussion

Participants experienced heat on three interconnected planes. The first is as an external climatic condition which is connected with measurements and scientific weather predictions. For example, when talking about the February 2009 heat wave one interviewee remarked:

*It's been very, very hot. It got to 44 or 45 degrees here. There were a few hot days*.

The second was people's embodied and subjective responses to heat expressed in such comments as *I find it enervating* or *I‘ve never worried about the heat*. In this dimension, we noted a lot of variation between couples with people living in the same environment responding to heat in different ways. The air-conditioner in particular was employed to manage these subjective responses to heat in a fairly unquestioning way, while others commented on its cost. Focus group participants in the aged-care facilities observed that they did not experience seasonal differences because the temperature inside remained constant and they rarely went outside. Air-conditioners are taken-for-granted, allowing embodied responses heat to be denied and ignored.

Third, the long history of Anglo-Australians’ relationship to heat can be seen in the way that heat is employed as a cultural metaphor signifying affinity with an archetypal Australian identity or personal resilience.

*I come from [an outback country town] – perhaps it's genetic [to not feel the heat]*.

This view was supported by people's stories about growing up with heat or accounts of when it was really hot in the past as opposed to more recent climatic conditions. Some participants said they were not bothered by heat as they had lived with it all their lives. This supports evidence that individuals become acclimatized to heat over long periods of time. These views were linked to a rejection of climate change and global warming on the basis that it was not really hotter than before. Individuals’ perceptions of heat over the course of their lives contradicted scientific evidence of warming as part of climate change.

Other factors that came into play when heat was discussed included time and financial flexibility. Participants were retired and none appeared to experience financial hardship. They were able to manage heat by undertaking activities in the cooler parts of the day in ways that people employed in 9 to 5 routines cannot or if they had the money and time they travelled to avoid temperature extremes. Overseas research points to the importance of poverty and poor social networks leading to the elderly losing social contact and not receiving medical attention. Even though they were not wealthy, participants had air-conditioned cars to travel in and used their phones to maintain social contact.

In Australia, it appears that new technologies are replacing everyday knowledge to deal with heat as people switch to home air conditioning and climate-controlled cars. Modern houses throughout much of Australia are regularly designed without eaves or verandas or with sealed windows to better suit air conditioning. Before air conditioning, people used their verandas extensively as they ‘created opportunities for casual observations and social exchange and people would eat, play and sleep outside’ (37). In contrast, air-conditioning has privatised comfort, drawing people from their social networks on the verandas back inside their homes. In the past, people would regulate the temperature, by stoking the fire or opening a window, personally evaluating what they considered to be comfortable displaying an achievement of personal skill and climatic awareness (35). Shove argues that the right to ‘comfort’ has become naturalised, normalised, and expected, reinforced and reproduced both by industry, architecture, and by the rituals of everyday life. As climate-controlled perfection can never be maintained naturally due to weather fluctuations, this construct has been profitable for the air conditioning industry. Between 1962 and 1992 American homes with air conditioning grew from 12 to 64%, (37: 49).

Most Australian families can turn on their home air conditioning or use their climate-controlled cars to drive to cooler outdoor destinations or retreat to air-conditioned shopping malls, cinemas, or libraries. During the 2009 Melbourne heat wave in the suburb of Boroondara, the four public pools increased in use by 90% compared to the same time the previous year where trips to aquatic centres proved very popular (38, 39). A Melbourne DVD shop manager, Glenn Sansome, said his store was extremely busy as customers stocked up on entertainment for the hot weather (40). Stores experienced a spike in sales of ice, water, ice-cream and cold drinks, air conditioners, fans, and other cooling appliances (41–43).

However, the use of such technologies comes at a considerable cost. One extreme case of state-wide energy failure occurred in the summer of 2004 in Western Australia forcing the Western Power company to ban the use of air conditioners in homes and offices and effectively ordering a shutdown of all industry. Western Power threatened to sue residents $1,000 and businesses $10,000 for non-compliance (44). Nevertheless, the following year, *The Australian* reported that ‘more than 100,000 new household air conditioners have been installed in Western Australia’ (45). While hospitals and aged-care facilities would be exempt from these restrictions, this study highlights the near universal reliance of older people on air-conditioning in their private homes. Such vulnerable, ‘free-living’ individuals may be at particular risk at these times.

Water is also crucial to Australians’ responses to heat. As we have noted, most Australians live near the coast. Even from the inland (Western) side of Sydney, the beaches are accessible for day trips and summer holidays. The coastal invasion can be seen as another example of Australians’ response to heat. However, Australia's more hospitable coastal regions along the Eastern and Southern seaboard and in Western Australia are rapidly disappearing under the pressure of urban development.

At an individual and community level, the importance of water as a ‘cooling device’ also holds dilemmas. Over the last decade or so in many Australian cities, severe water restrictions were imposed on households as dam levels dropped dramatically. Individuals were exhorted to reduce their water consumption making the use of showers and baths for cooling less accessible. In many places, these once temporary restrictions have now become permanent. At the community level, public swimming pools were used very heavily during heat waves providing an important and widely accessible community resource that have many benefits beyond cooling. As water stocks become increasingly scarce and expensive with climate change, this ‘solution’ will become less accessible. However, increasing water scarcity and restrictions on its use is already proving to be an important and effective mobilizer of Australian concerns about climate change than heat.

This small study leaves many issues unexplored. We need to plan for disadvantaged groups who cannot afford air-conditioned houses and cars during heat waves in Australia, and have systems in place for when power fails or is restricted during extreme heat. As Australia's population ages, an increasing proportion of people will become more vulnerable to the effects of heat. Young children are another physiologically vulnerable group and thus far we do not know how parents manage them during heat waves and what additional pressures it places on them. Further research should also examine how attitudes to climate change intersect with socio-economic and urban–rural differences because as Nightingale (46) observes, adaptation to climate change is ‘a dynamic process that brings together social inequalities, power, knowledge, geo-politics and day-to-day interactions’.

## Conclusion

Embodied experiences of heat among our interviewees were variable, inconsistent, and individualistic even among couples living in the same dwelling, demonstrating how difficult it is to orchestrate a consistent population level response to climate change with the support of individuals. The current Australian response to heat is to rely on air-conditioners with obvious impacts on carbon emissions (most of Australia's electricity is derived from coal) thus fostering climate change conditions that contribute to heat waves (47). Overall, modern technologies, tastes, and house designs elevate energy consumption, ensuring a further barrier to climate change mitigation and increasing the importance of climate adaptation.

Understanding the positive and negative ways in which heat, and the experience of heat, can be represented culturally will be important for Australian society's willingness to change practices to reduce carbon emissions. From a sustainability perspective, Lowe (7) suggests that our cultural background has informed the slow uptake of Australians to use solar energy, while Hamilton (48) believes our cultural character has fostered a state of denial to act on climate change. Safeguarding the health of Australians through the use of technologies and water may not be sustainable leading to more damaging secondary and tertiary health effects in the future.
